# Bubonic Plague—Malignant Polyadenitis

**Published:** 1897-05

**Authors:** Walter Wyman

**Affiliations:** Surgeon-General, U. S. Marine-Hospital Service, Washington, D. C.


### Bubonic Plague—Malignant Polyadenitis.

BY WALTER WYMAN, A. M., M. D.,
Surgeon-General, U. S. Marine-Hospital Service, Washington, D. C.

Extracted from the American Journal of the Medical Sciences, March, 1897.

Our modern knowledge of plague is due chiefly to the efforts and writings of Kitasato, Aoyama, Yersin, Wilm, Cantlie, Lowson and Arnold.

Kitasato and Aoyama, with assistants, were commissioned by the Japanese Government to study the plague in Hong-Kong in 1894—Kitasato to make bacteriological investigation, and Aoyama to report upon the clinical and pathological characteristics of the plague.

Kitasato discovered the plague-bacillus, and first published the results of his investigations under the auspices of the University of Tokio, July 7th, 1894. His report was reproduced in the *British Medical Journal*, and may also be found in full in the *Annual Report of the Marine-Hospital Service* for 1894.

Aoyama published his report in the *Mittheilungen aus der Medicinischen Facultat der Kaiserlich-Japanischen Universitat*, Tokio, June, 1895.

Yersin was commissioned by the French Minister for the Colonies to investigate the plague at Hong-Kong, and conducted his researches in 1894, both in Hong-Kong and Canton. The results were published in the *Annals of the Pasteur Institute*, September, 1894.

A second article in the *Annals of the Pasteur Institute*, July, 1895, was published jointly by Yersin, Calmette, and Borrel, as the result of investigations in the laboratory of Roux in the Pasteur Institute. This article relates chiefly to the study of the toxins and the serum-therapy of the disease.

In January, 1896, Yersin, under the auspices of the French Government, returned to Annam (Cochin-China), and established there (at Nha Trang) a Pasteur Institute for the investigation of the disease.

In June, the same year, he proceeded to Canton, where he treated successfully a case of plague with serum brought from the Pasteur Institute at Nha Trang, this being the first case of plague subjected to the serum-therapy. He then proceeded to Amoy, where he treated twenty-three cases of plague, with twenty-one recoveries, with serum brought from the Pasteur Institute in Paris. The details of the above work are published in a third article by Yersin in the *Annals of the Pasteur Institute*, January 25, 1897.

Dr. Wilm, a surgeon of the German navy, was commissioned by his government to make an investigation of the pest in Hong-Kong during the epidemic in 1895, and made report thereon May 20, 1806.

Cantlie (lecturer on applied anatomy, Charing Cross Hospital Medical School, late of Hong-Kong) had opportunity for studying and recording the disease during the Hong-Kong epidemic, and a very complete lecture thereon is published in the London *Lancet* of January 2 and 9, 1897.

James A. Lowson, medical officer in charge of the Epidemic Hospital in Hong-Kong, has made a full report upon the epidemic of 1894, published by Noronha & Co., government printers, Hong-Kong, 1895.

W. F. Arnold, Passed-Assistant Surgeon, U. S. Navy, studied the plague during its exacerbation in Hong-Kong in 1896. A preliminary report is published in the *Annual Report of the Surgeon-General of the U. S. Navy* for 1896. It is to this observer that we are indebted for the cultures which form the basis of the experiments now being conducted in three laboratories in the United States.

DEFINITION.—Plague, or malignant polyadenitis, is an acute febrile disease, of an intensely fatal nature, characterized by inflammation of the lymphatic glands, marked cerebral and vascular disturbances, and by the presence of a specific bacillus. Although one gland alone may be clinically apparent, most, if not all, of the lymphatic glands are found to be enlarged at the post-mortem examinations. (Cantlie.)

PERIOD OF INCUBATION.—Three to five days, possibly eight (Kitasato); four and one-half to six days (Yersin); two to seven days (Aoyama); three to six days (Cantlie); may reach nine days (Lowson).

Cantlie adds: "I do not think that we have evidence to place the period of incubation of malignant polyadenitis beyond the end of the sixth day after exposure. Be it observed, however, thii extends only to plague-infection during an epidemic. The evidence to hand the last few weeks from Calcutta and from London renders the period of incubation of ambulatory plague uncertain."

SYMPTOMS AND DURATION OF THE DISEASE.—"The patient complains of high fever and swelling of one or more of the lymphatic glands. These swellings may antedate, coincide with, or follow the rise in temperature, and are accompanied by severe pain. The most common gland affected is one of the femoral chain, next an inguinal, next axillary, and sometimes a cervical gland is affected. The tongue is coated with a grayish white or dark-brown heavy fur. There is commonly headache, also delirium; the heart is generally affected; occasionally vomiting and diarrhœa are present (not frequently the last two conditions, which are generally forerunners of a fatal issue).

"In patients who survive the outset of the disease the temperature does not fall until a week has passed, and convalescence is a slow process.

"If in such a case as described the blood be examined, the before-described bacilli, in greater or less numbers, will be found present.

"It is not always an easy matter to demonstrate the presence of the bacilli directly in the blood of many patients; they are present sometimes in such small numerical strength that only after examining several slides can they be discovered. In order to be safe not only must the blood of a suspected plague patient be examined, but a cultivation should also be made.

"In the buboes the bacilli always occur in the form of pure cultivations, but it is obviously not always easy to procure a specimen of bubo-contents from the living subject." (Kitasato.)

"When prodromal symptoms occur they generally last not more than two hours, and include lassitude, headache and malaise, vomiting, loss of appetite, dizziness, pain in the limbs, gland-swelling. The temperature soon reaches 39° or 40° C., or even higher, and continues high.

"Delirium occurs very early in the attack. It is seldom violent. The face is at first very red. Pulse strong, tense and dicrotic. Urine dark red and frequently albuminous.

"The gland affection occurs as the fever begins to rise; the swelling progresses rapidly, and in two days reaches the size of an egg. The pain is often so intense as to extort cries of anguish from the patient. At other times the swelling can only be detected by pressure, and is not painful. The swelling disperses or gathers in a huge collection of pus. Dispersion is slow, and often the swelling is perceptible for two months.

"The period of recovery begins usually with the end of the first or the beginning of the second week. Complications are numerous, namely, nephritis, abscesses, retention of urine, icterus, carbuncle, etc."

"Death usually occurs at the height of the disease, and between the second and eighth day. Intermissions in the fever do occur. (Lawson.) Death seldom occurs later than between the second and eighth day." (Aoyama.)

Yersin describes the course of the disease as follows: Overwhelming prostration. The patient is suddenly seized with a high fever, accompanied by delirum. After the first day a bubo, usually single, appears. In 75 per cent of the cases this bubo was in the groin, in 10 per cent in the axilla—rarely in the neck or other regions. The gland affected quickly attains the size of a hen's egg. Death supervenes at the end of forty-eight hours, and frequently sooner. When life is prolonged for five or six days the prognosis is better. The gland then softens, and we may then operate and evacuate the pus. In many cases the bubo has not time to form, and then we have hemorrhages from the mucous membrane and petechial spots on the skin. The mortality is very large.

Wilm states that in one to five or six days after the fever begins the glands begin to swell. The duration of the fever is usually from a few hours to some weeks. In about 30 per cent of the recorded cases the fever of the infection lasted about five or six days, and this may be regarded as typical. It is high at the beginning and sinks slowly, with frequent returning remissions. It may last as long as ten days. After this primary fever a secondary fever occurs in a majority of recovering cases. This is the "fever of absorption," and may lead to weeks of exhausting illness, and cases may die at this stage which were saved in the battle of infection.

As to the swollen glands, Wilm says: "Even large buboes may form in a few hours after a time when a person has felt absolutely in the best of health. On the other hand, we frequently see a patient dying of plague without being able to feel a single affected gland, and only a thorough post-mortem examination shows the slightly swollen glands of lentil, pea, or almond size, which yields the plague bacillus by microscope and culture tube." 76 per cent of Wilm's observed recorded cases died in the first six days. Death was generally caused by paralysis of the heart; in other cases it was from the brain complication (meningitis, cerebritis and hemorrhage). The temperature at death is sometimes very high; sometimes subnormal. Convalescence is generally prolonged and often complicated with suppurative fever.

Death-rate.—(From official reports, Hong Kong, 1894—Lowson.)—Chinese, 93.4 per cent; Indians, 77 per cent; Japanese, 60 per cent; Eurasions, 100 per cent; Europeans, 18.2 per cent. Lowson adds: "No doubt the European blood and stamma had a good deal to do with the recovery—this, notwithstanding the more careful nursing they received. Early treatment and confidence in the European medical attendant were also in their favor."

Treatment.—According to Tyson, free stimulation, nutritious food, together with cool baths to combat the fever, are the measures indicated. Antiseptic treatment of the abcesses should be praticed, and may shorten the duration of these plagues of the skin as compared with the older treatment.

Kitasato's general directions relate to hygienic measures; proper recaptacles for sewerage, purity of water supply, isolation of the sick, disinfection of clothing and bedding and of the evacuations, all contact with the sick to be avoided, great care to be exercised with regard to food and drink, and after recovery the patient to be kept in isolation at least for a month.

Lowson advises: "Never use depressants if you can possibly do without them," and states that brandy and tepid sponging were without doubt the best antipyretics.

The serum-therapy, as demonstrated by Yersin, is highly efficacious. Thirty cubic centimeters of the serum from an immunized horse cured the first severe case of plague upon which it was tried. Yersin in ten days, in Amoy, treated twenty-three cases of plague. Almost all of them were treated in Chinese houses under bad sanitary conditions. His results were as follows:

Six plague-cases taken on the first day of the disease. Cure obtained in all from twelve to twenty-four hours, without suppuration of the bubo, by injection of from 20 to 30 c.cm. of serum.

Six taken on second day. Cure slower; 30 to 50 c.cm. of serum used. Complete cure without suppuration in three or four days.

Four taken on third day. Fever persisted one or two days after commencement of injections. Cure was slower, and in two cases the buboes suppurated. Serum injected, 40 to 60 c.cm.

Three taken on furth day. Cured in five or six days. One bubo suppurated. Serum injected, 20 to 50 c.cm.

Four taken on fifth day. Two died, whose cases were desperate from the outset. Serum used, 60 to 90 c.cm.

To the date of Yersin's report 26 plague cases had been treated with serum—3 at Canton, 23 at Amoy. Of these only two died.

Varieties of the Disease.—The preceding compilation relates to "malignant polyadenitis," which embraces both forms of plague known as "fulminant" and "typical."

According to Cantile, the varieties of plague are (a) fulminant, (b) typical, (c) pestis minor, and they are all allied. He suggests that an appropriate name for the fulminant and typical plague is "malignant polyadenitis," and an appropriate name for the mild variety (pestis minor) "benignant polyadenitis."

A benign polyadenitis, according to Cantile, may run its course without being preceded or followed by the malignant variety, and the malignant polyadenitis may run its course without being preceded or followed by the benigh variety, yet typical plague —malignant polyadenitis—is frequently associated with pestis minor—benign polyadenitis. A bacillus of somewhat similar appearance microscopically is reputed to be found in both. The cause of fulminant and typical plague is a diplo-bacterium in the blood and tissues. The cause of pestis minor, or benignant polyadenitis, may be an allied diplo-bacterium, but with less toxic power.

Pestis minor, until within a short time, according to Cantile, was considered a disease of wholly different nature from that of true plague. Recently, however, news has been received from Calcutta of the discovery of a bacillus allied to the Kitasota bacillus of true plague, and associated with the variety of plague known as pestis minor. In this disease the temperature is rarely high, although it has been known to be 104° F. The duration is from ten to twenty days usually, but may be eight weeks, for most of which time the general health is but little impaired and the patient able to go about as usual. It may be observed here that the true relation of pestis minor to the true plague has not been satisfactorily determined. Cantile concludes that pestis ambulans may become a malignant polyadenitis in the same person, even although the patient is removed from the infected zone, and that the period of incubation of ambulatory plague is uncertain. This is based upon the history of a case of a lad seventeen years of age, who had been exposed to plague in Bombay, and fifteen days before leaving Bombay for Calcutta had noticed swellings first in one groin and then in another, but never felt ill until his arrivel in Calcutta. He was examined, and a diplo-bacterium identical with the Kitasato bacillus was found in his blood, and the clinical symptoms of plague were manifest. It is an unpleasant reflection that a person may be infected with plague fifteen or twenty days without feeling any inconvenience, but at the expiration of that time have the disease appear in its virulent form.

Plague-bacillus.—As the report of Kitasato published in English is readily available, and inasmuch as I have seen no translation of the article of Yersin, the limits of this paper preventing the observations of both writers, I insert here a translation of Yersin's description of the bacillus, taken from the *Annals of the Pasteur Institute*, September, 1894.

"The first indication was to see if a microbe existed in the blood and in the pulp of the buboes of those sick with the disease. The pulp of the buboes is filled in all cases with a veritable puree of a short, non-motile bacillus, with rounded ends, staining easily with the ordinary aniline dyes, but not staining with Gram's method. The poles of the bacillus are stained more deeply than the center, so that often a clear space is seen in the middle of the rod. Sometimes the bacillus appears to be surrounded by a capsule. It is found in great numbers in all the buboes and glands of patients. The blood also contains it sometimes, but in much smaller numbers; it is there only in very grave cases and those rapidly fatal.

"The pulp of the buboes planted upon gelatin gives rise to the development of white transparent colonies, presenting iridescent margins when viewed by reflected light. Culture is best made in glycerin-agar. The bacillus also grows upon coagulated blood-serum. In bouillon the growth presents a very characteristics appearance, quite similar to that of erysipelas—a clear liquid with grumous deposits along the walls and at the bottom of the tube. An alkaline solution of peptone, 2 per cent., with the addition of 1 to 2 per cent. of gelatin, is the most favorable medium. These cultures examined under the microscope show true chains of bacilli; sometimes presenting bulbous enlargements. Upon the gelatin, if we examine with great care and with a high magnification, we find among the normal forms bacilli sometimes deformed and sometimes chains formed of rods disposed laterally. These deformed and abnormal forms become more and more numerous in old cultures, and they stain but poorly."

According to Zetthor and Koch, as quoted in the London *Lancet*, February 6th, the bacillus is a "bacillus asporigenus," which dies after four days, during which it is kept at a dry heat, or at a temperature of 80° C. for half an hour, or at that of 100° C. for a few minutes. Its resisting power to chemical disinfectants is feeble, succumbing shortly in a 1 per cent. solution of carbolic acid or of lime water. On the other hand, it develops easily in many culture-media at the ordinary temperature (from 18° to 22° C.), having been found in the soil and in the dust of houses where those ill from it, or who had died from it, have been lodged.

DISINFECTION EXPERIMENTS UPON THE BACILLUS OF BUBONIC PLAGUE.

| | 5 minutes. | 10 minutes. | 15 minutes. | 30 minutes. |
|---|---|---|---|---|
| Formaldehyd: | | | | |
| 1 : 1000 | No growth. | No growth. | No growth. | |
| 1 : 2000 | Growth. | Growth. | No growth. | |
| 1 : 3000 | Growth. | Growth. | Growth. | |
| Carrosive sublimate: | | | | |
| 1 : 1000 | No growth. | No growth. | No growth. | |
| 1 : 2000 | No growth. | No growth. | No growth. | |
| 1 : 3000 | No growth. | No growth. | No growth. | |
| 1 : 5000 | Growth. | No growth. | No growth. | |
| 1 : 10,000 | | No growth. | No growth. | |
| 1 : 15,000 | | | No growth. | No growth. |
| Lime: | | | | |
| Saturated solution | | Growth. | No growth. | No growth. |
| Carbolic acid: | | | | |
| 0.25 per cent. | Growth. | Growth. | Growth. | |
| 0.50 " | Growth. | Growth. | Growth. | |
| 1.00 " | No growth. | No growth. | No growth. | |
| Trikresol: | | | | |
| 0.25 per cent. | No growth. | No growth. | No growth. | |
| 0.50 " | No growth. | No growth. | No growth. | |
| 1.00 " | No growth. | No growth. | No growth. | |
| Sulphur dioxide: | | | | |
| 1.25 per cent. | | No growth. | | |
| 2.20 " | | No growth. | | |
| 5.00 " | | No growth. | | |
| 10.00 " | | No growth. | | |

Thermal death-point between 58° and 60° C.

So far as known to the writer, experiments with the plague-bacillus are being conducted in but three laboratories in the United States, namely: Hoagland Laboratory in Brooklyn, the Laboratory of the Health Department of New York City, and the Laboratory of the Marine Hospital Service in Washington. It may be said, in general, that the investigations in these laboratories are confirmatory of the results of the investigations in the laboratories heretofore mentioned. A special investigation is being conducted, however, in the Hoagland Laboratory by Dr. E. H. Wilson, upon the life-history of the bacillus, and in a telegram received February 14th, in response to an inquiry, he informs me that the bacillus in a dark closet, on filter-paper and on wool-blanket, is still living at the end of the eighteenth day. The experiments conducted by Drs. J. J. Kinyoun and H. D. Geddings in the Laboratory of the Marine Hospital Service have related especially to the action of disinfectants, the results of which are set forth in the preceding table.

The prescribed limits of this article prevent any considerations concerning the contagious and infectious character of the disease and the conditions which cause its spread. Neither is it possible herein to describe the measures which have been taken to prevent the spread of the disease in foreign countries, and to prevent its introduction into the United States. These subjects would require a special treatise.

### Therapeutic Notes.

Dr. Edlin, of Illinois, recommends the following for consumption:

℞ Creoscti fagi..........................℥ j,

Guaiacol................................ʒ iv.

Alcoholis................................℥ iv.

Glycerini................................℥ villj.

Ex. aurantii fl..........................℥ ij.

Saccharini...............................gr. xv.

Vini xerici..................q. s. ad O ij.

M. Sig.: Two drachms in a glass of water after meals. Increase one drachm a day up to one ounce at a dose.

Dr. Edlin says: This solution has given me more satisfaction than any other preparation of the kind. The taste is not very repugnant. Digestive disturbances are rare. The bowels become regular. Appetite is increased, and a general feeling of well-being is induced in a short time. The glycerin seems to be a valuable agent in this preparation, as it is both a food and a regulator of the bowels. The amount of alcohol is not large enough to disturb digestion, but is sufficient for stimulation. Furthermore, this solution is less apt to injure the action of the kidneys. In only two cases of far-advanced tuberculosis with chronic kidney disease has it disagreed. On the whole, it seems to have greater effect on the disease than when creosote or guaiacol is given undissolved, or in pills. The various preparations of these drugs are of inferior value, as their therapeutic action is weakened. Another objection to their use is their costliness. Creosote and guaiacol are nearly specific in scrofula, and a cure is sooner effected with them than with any other drug. No other medicine has in my hands been of greater service in this disease.—*N. Y. Journal*, April 17th.

---

**A Chilly Day for Willie.**—The Reverend Dean Hole, of Rochester, England, in his little book, "A Little Town in America," says that he picked up the following bit of posey in Cincinnati:

Little Willie from his mirror
  Sucked the mercury all off,
Thinking, in his childish error,
  It would cure his whooping-cough.
At the funeral Willie's mother
  Smartly said to Mrs. Brown,
"'Twas a chilly day for William
  When the mercury went down."

—*Jour. Amer. Med. Ass'n.*

---

### Arsenic in the Treatment of Asthma.

Dr. WilliamMurray (*Rough Notes on Remedies*, London, 1897, *Medical Chronicle*, March, 1897), says the following prescription has achieved the happiest results in spasmodic asthma:

℞ Tincture of stramonium.............. 2 drachms.
Ammonium carbonate............... 1 drachm.
Sodium bicarbonate.................... 3 drachms.
Magnesium carbonate................ 1 drachm.
Powdered rhubarb.....................20 grains.
Chloroform.............................20 minims.
Peppermint water, enough to
  make....................................8 ounces.

M. Sig.: Half an ounce to be taken three times a day with an ounce of water.

"Having thus secured a temporary lull in the complaint," says the author, "the patient must be put on a course of arsenic, care being taken to give just as much as the stomach will bear. A good plan is to give Fowler's solution—five drops—with breakfast and dinner, and maintain the corrective dose with stramonium at night."

